# Data in support of alpha1beta1 and integrin-linked kinase interact and modulate angiotensin II effects in vascular smooth muscle cells

**DOI:** 10.1016/j.dib.2015.11.053

**Published:** 2015-12-17

**Authors:** João Alfredo Moraes, Ana Clara Frony, Aline Maria Dias, Mariana Renovato-Martins, Genilson Rodrigues, Cezary Marcinkiewicz, Jamil Assreuy, Christina Barja-Fidalgo

**Affiliations:** a Laboratory of Cellular and Molecular Pharmacology, Department of Cell Biology, IBRAG, Universidade do Estado do Rio de Janeiro (UERJ), Rio de Janeiro, RJ, Brazil; b Department of Bioengineering, College of Engineering, Temple University, Philadelphia, PA, USA; c Department of Pharmacology, Universidade Federal de Santa Catarina (UFSC), Florianópolis, SC, Brazil

## Abstract

The data provides information in support of the research article Moraes et al., Atherosclerosis 243(2) (2015) 477–485 [1]. Here we provide data behind the mechanisms involved in Angiotensin II (Ang II) effects on vascular smooth muscle cells (VSMC). Ang II-induced VSMC ROS production is modulated by alpha1beta1 integrin. Ang II also stimulates ROS production in VSMC *via* p47^*phox*^, a NOX2 subunit. Furthermore, Ang II effect on VSMC migration was also inhibited by NOX2 inhibitor. We showed that obtustatin, alpha1beta1 integrin blocker, inhibited Ang II effect on p47^*phox*^ activation. Ang II effect on ROS production is also PI3K dependent. Finally we showed that NOX1 and Integrin-Linked-Kinase (ILK) are crucial to NOX2 activation. The research provides information about the sequential events of NOX1/alpha1beta1 integrin/ILK/NOX2 in Ang II effects on VSMC.

## Specifications Table

**Table:** 

Subject area	*Biology*
More specific subject area	*Cardiovascular pharmacology; Cellular pharmacology*
Type of data	*Table and figure*
How data was acquired	*Boyden chamber, Microscope, Plate reader (Envision®), Western blotting*
Data format	*Raw, filtered, analyzed, etc*
Experimental factors	*Effect of Angiotensin II on vascular smooth muscle cells*
Experimental features	*Role of NOX1/2-derived reactive species oxygen, alpha1beta1 integrin and Integrin linked-kinase*
Data source location	*Rio de Janeiro, Brazil*
Data accessibility	*The data presented in this article and is related to*[1]

## Value of the data

• These data provides a thorough understanding the molecular events underlying Angiotensin II effect on vascular smooth muscle cells.
• The data shows dual-NOX involvement in Angiotensin II effect on vascular smooth muscle cell: a rapid NOX1 activation which is accompanied by NOX2.
• The data shows that Angiotensin II effect on NOX2 activation depends on NOX1 and ILK.
• These data also show that NOX2 is involved in Angiotensin II effect on vascular smooth muscle cell migration.

## 1. Data

### 1.1 Alpha1beta1 modulates the second interval of Ang II-induced ROS production in VSMC

Table 1 shows the detailed analysis of ROS production showed in Fig. 1B of Ref. [1]. This table shows that ROS production induced in VSMC occurred in two stages, and that obtustatin inhibited only the second part (0.5–1 h) of ROS production.

### 1.2 Time-course of angiotensin II-induced ROS production and its dependence on NOX2 in VSMC

**Fig. 1:**
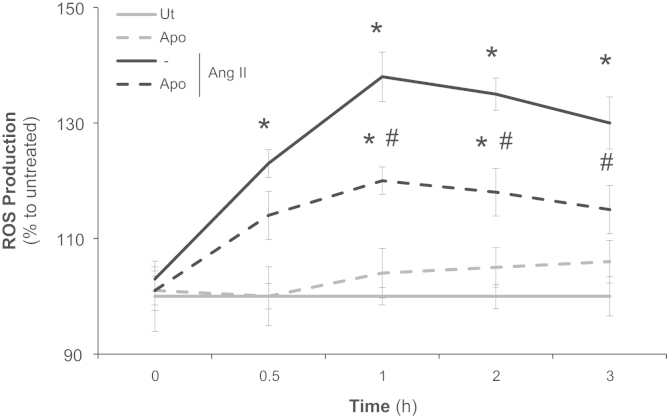
Time-course of angiotensin II-induced ROS production and its dependence on NOX2 in VSMC. ROS production (assessed by CM-H_2_DCFDA) was evaluated in primary VSMC incubated with angiotensin II (Ang II) 100 nM. Apocynin 10 µΜ was added 15 min before Ang II. The results are representative of three independent experiments. Data are expressed as means±SD. *p<0.05 *vs*. untreated; #*p*<0.05 *vs*. the treated with Ang II.

Apocynin, a selective inhibitor of p47^*phox*^ (a NOX2 subunit), inhibited only the second peak of ROS (0.5–1 h) production induced by Ang II in VSMC (Fig. 1).

### 1.3 Angiotensin II induces VSMC migration *via* NOX2

**Fig. 2:**
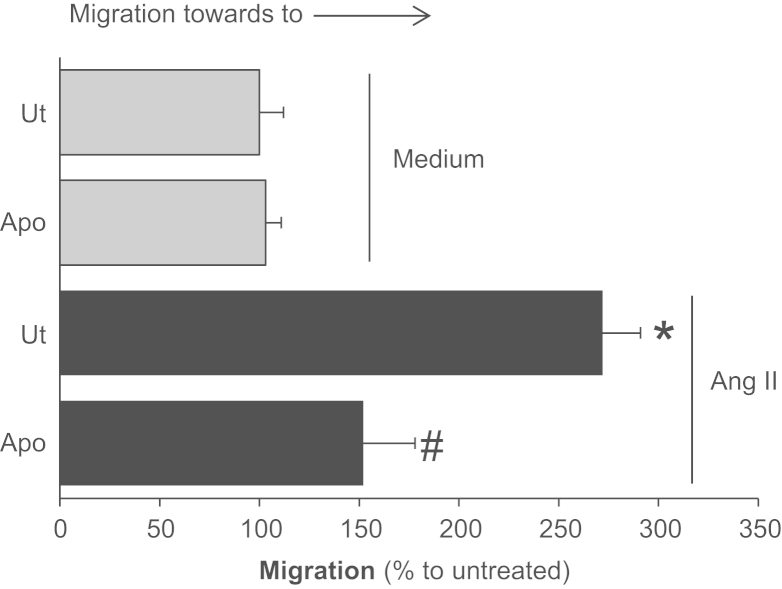
Angiotensin II induces VSMC migration *via* NOX2. Primary VSMC were incubated for 15 min with apocynin 10 µM and cells were placed in modified Boyden chemotaxis chambers towards an angiotensin II (Ang II) 100 nM gradient for 4 h at 37 °C. The results are representative of three independent experiments. Data are expressed as means±SD or SD. *p<0.05 *vs*. untreated; #*p*<0.05 *vs.* the treated with Ang II.

Ang II effect on VSMC migration also relies in NOX2 activity, once we observed that the pretreatment of VSMC with Apocynin inhibited the chemotactic effect of Ang II (Fig. 2).

### 1.4 A7r5 contains NOX1, NOX2 and NOX4

**Fig. 3:**
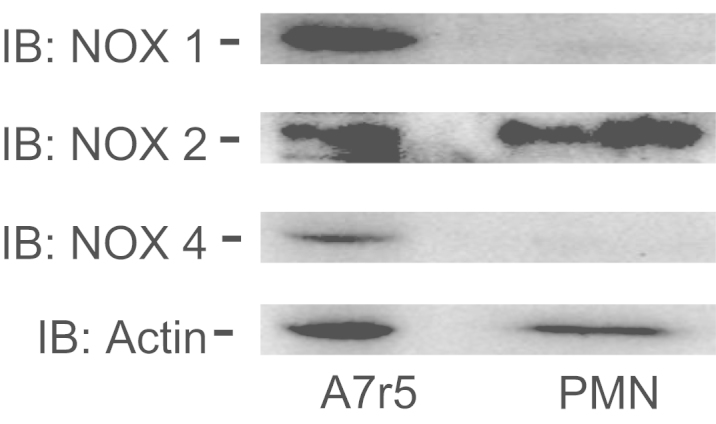
A7r5 contains NOX1, NOX2 and NOX4. A7r5 cells and human polymorphonuclear neutrophils were lysed and subjected to SDS-PAGE and blotting for NOX1, NOX2, NOX4 and β-actin (load control) proteins. The results are representative of two independent experiments.

It is well described that VSMC contains NOX2 [2], [3], so we investigated the presence of this protein in the VSMC line A7r5. In our data we observed that besides NOX1 and NOX4, A7r5 also has NOX2 at similar levels compared to polymorphonuclear neutrophils (Fig. 3).

### 1.5 p47^*phox*^ and alpha1beta1 integrin modulate angiotensin II effect on NOX2 activation in VSMC

**Fig. 4:**
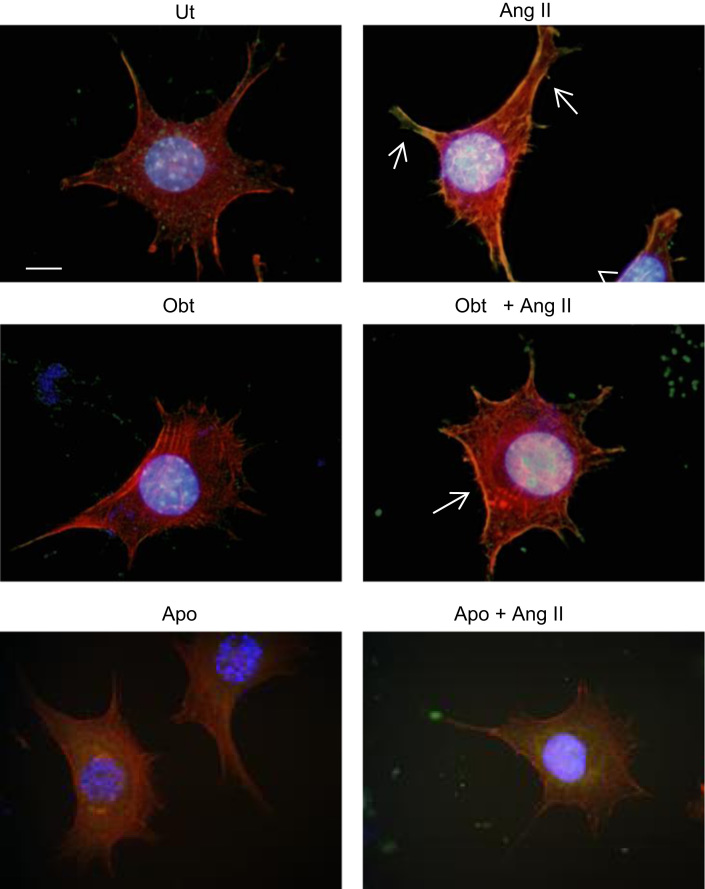
p47^*phox*^ and alpha1beta1 integrin modulate angiotensin II effect on NOX2 activation in VSMC. Primary VSMC obtained from rat aorta untreated (top, left); treated with obtustatin (obt) 100 nM for 1 h (bottom, left); stimulated with Ang II 100 nM for 1 h (top, right), or treated with obtustatin (100 nM) and stimulated with Ang II (bottom, right). p47^*phox*^ membrane translocation was evaluated by immunofluorescence staining with anti-p47^*phox*^-Alexa 388 (green), actin was visualized through phalloidin-rodhamin (red) and nuclei was visualized through DAPI (blue). Scale bar: 10 μm. White arrows indicate p47^*phox*^ presence in cell membrane (yellow). Result is representative of three independent experiments.

When p47^*phox*^ interacts with the NOX2 membrane subunit, NOX2 becomes a full complex and it is ready to initiate ROS production [4], [5]. Fig. 4 shows that Ang II induced p47^*phox*^ translocation to plasma membrane and that apocynin and obtustatin inhibited this effect, indicating a dependence on p47^*phox*^ and alpha1beta1 integrin for Ang II-induced NOX2 activation.

### 1.6 Angiotensin II induces p47^*phox*^ activation in VSMC

**Fig. 5:**
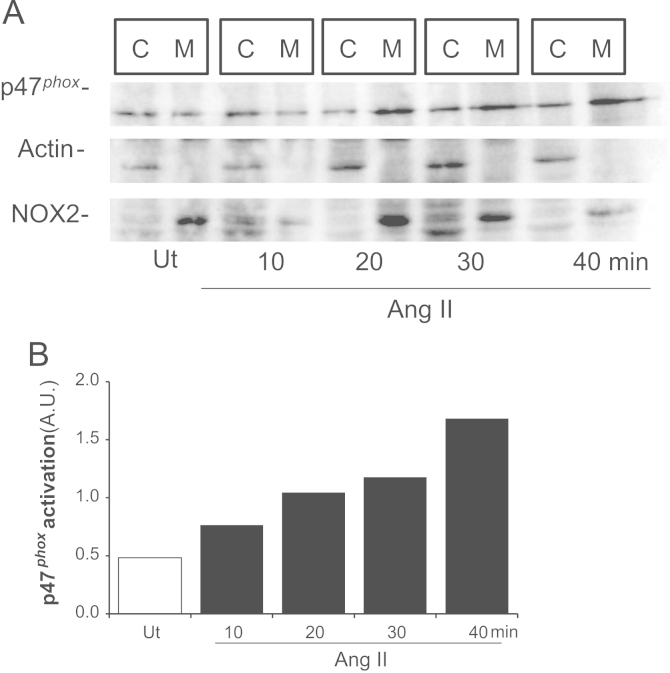
Angiotensin II induces p47^phox^ activation in VSMC. Primary VSMC were treated with Angiotensin II (Ang II) 100 nM for the indicated times at 37 °C/5% CO_2_, lysed and subjected to ultracentrifugation at 40,000*g* for 1 h. A. Cytosolic (C; the supernatant) and membrane (M; the pellet) fractions (10 µg/lane) were subjected to SDS-PAGE and blotting for p47^*phox*^, β-actin (cytosolic load control), and NOX2 (membrane load control) proteins. B. p47^*phox*^ activation was measured by p47^*phox*^/NOX2 membrane ratio. The results are representative of two independent experiments.

As we can observe through cytosol/membrane fraction analysis, Ang II was able to induce p47^*phox*^ translocation to VSMC membrane 10 min after treatment (Fig. 5A). Densitometry can be observed in Fig. 5B.

### 1.7 Angiotensin II induces ROS production *via* PI3K/AKT in VSMC

**Fig. 6:**
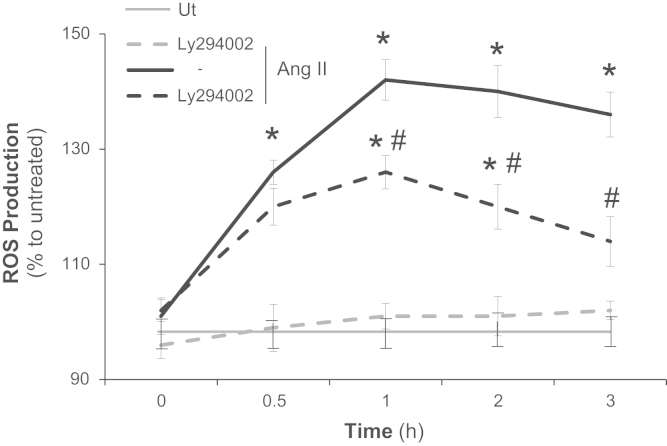
Angiotensin II induces ROS production *via* PI3K/AKT in VSMC. Primary VSMC were incubated with LY294002 (50 μM) for 15 min at 37 °C/5% CO_2_. Angiotensin II (Ang II) 100 nM was added and ROS production was followed up to 3 h. Results are representative of three independent experiments. Data are expressed as mean±SD. *p<0.05 *vs*. untreated; #*p*<0.05 *vs*. the treated with Ang II.

The treatment of VSMC with LY294002, a PI3K-AKT inhibitor, impaired the second peak of ROS production (after 30 min) induced by Ang II (Fig. 6).

### 1.8 siRNA silencing in VSMC

**Fig. 7:**
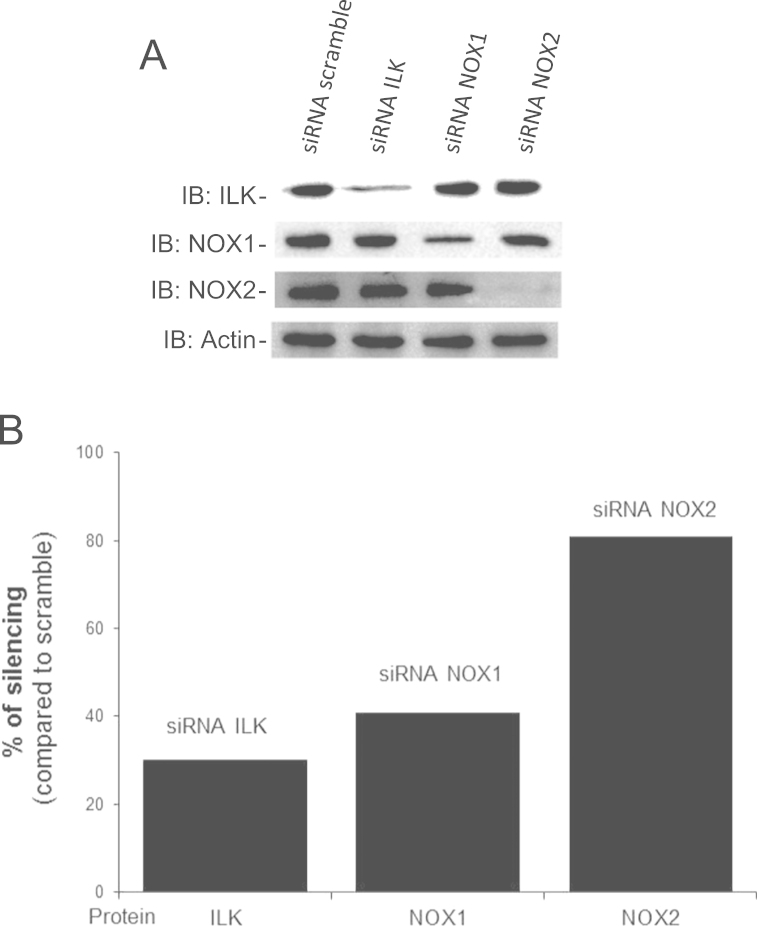
siRNA silencing in VSMC. A7r5 cells were transfected with siRNA to NOX1, NOX, ILK or siRNA scramble for 24 h. All treatments were performed at 37 °C/5% CO_2_. A. Whole cell extracts were prepared and subjected to Western blotting for ILK, NOX1, NOX2 and β-actin (load control). B. Effect of each siRNA on its respective target was quantified and it was showed in % of silencing.

For the set of experiments using siRNA technique we used A7r5 rat aorta smooth muscle cell line, which exhibit similar responses to Ang II when compared to primary VSMC (data not shown). Fig. 7A shows the western blotting of whole cell lysate of cells transfected with the specific siRNA (ILK, NOX1 and NOX2) and blotted to ILK, NOX1, NOX2 and actin. Specific siRNA treatment induced, respectively, 50% inhibition in ILK and NOX1 expression and almost 80% reduction of NOX2 (Fig. 7B). It was not possible to completely silence ILK and NOX1 expression since it caused cell death (data not shown).

### 1.9 ILK modulates the second interval of Ang II-induced ROS production in VSMC

Table 2 shows that Ang II was not able to induce any peak of ROS production in A7r5 silenced cells to NOX1. Furthermore silencing to ILK just interfered with the second peak of ROS production induced by Ang II.

### 1.10 NOX1 and integrin linked kinase (ILK) modulate angiotensin II effect on NOX2 activation in VSMC

**Fig. 8:**
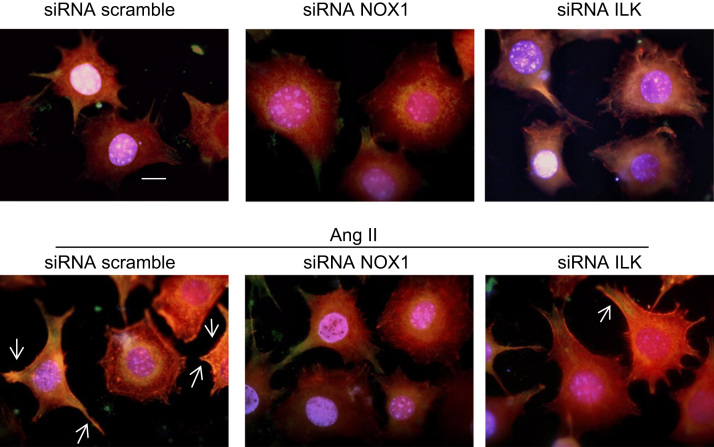
NOX1 and integrin linked kinase (ILK) modulate angiotensin II effect on NOX2 activation in VSMC. A7r5 cells were transfected with siRNA to NOX1, ILK or siRNA scramble for 24 h. All treatments were performed at 37 °C/5% CO_2_. A7r5 cells were stimulated with Ang II 100 nM for 1h. p47^*phox*^ membrane translocation was evaluated by immunofluorescence staining with anti-p47^*phox*^-Alexa 388 (green), actin was visualized using phalloidin-rodhamin (red) and nuclei was visualized using DAPI (blue). Scale bar: 10 μm. White arrows indicate p47^*phox*^ presence in cell membrane (yellow). Result is representative of three independent experiments.

Ang II was not able to induce NOX2 activation in A7r5 silenced cells to ILK or NOX1. On the other hand, Ang II was able to activate NOX2 in A7r5 transfected with siRNA scramble (Fig. 8).

### 1.11 Alpha1beta1 integrin does not modulate Angiotensin II effect on mitochondrial ROS production in VSMC

**Fig. 9:**
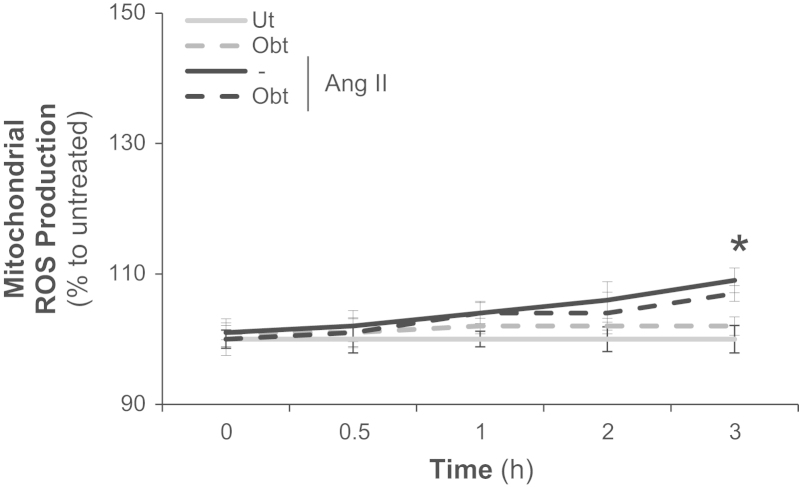
Alpha1beta1 integrin does not modulate Angiotensin II effect on mitochondrial ROS production in VSMC. Primary VSMC were incubated with obtustatin (Obt) 100 nM for 15 min at 37 °C/5% CO_2_. Angiotensin II (Ang II) 100 nM was added and mitochondrial ROS production (assessed using MitoSox, an indicator of mitochondrial ROS production) was followed up to 3 h. Results are representative of three independent experiments. Data are expressed as mean±SD. *p<0.05 *vs*. untreated; #*p*<0.05 *vs*. the treated with Ang II.

Ang II induced mitochondrial ROS production in VSMC after 3 h of treatment. We also observed that this effect was not alpha1beta1 integrin dependent, once obtustatin was not able to inhibit Ang II effect (Fig. 9).

### 1.12 Proposed model

**Fig. 10:**
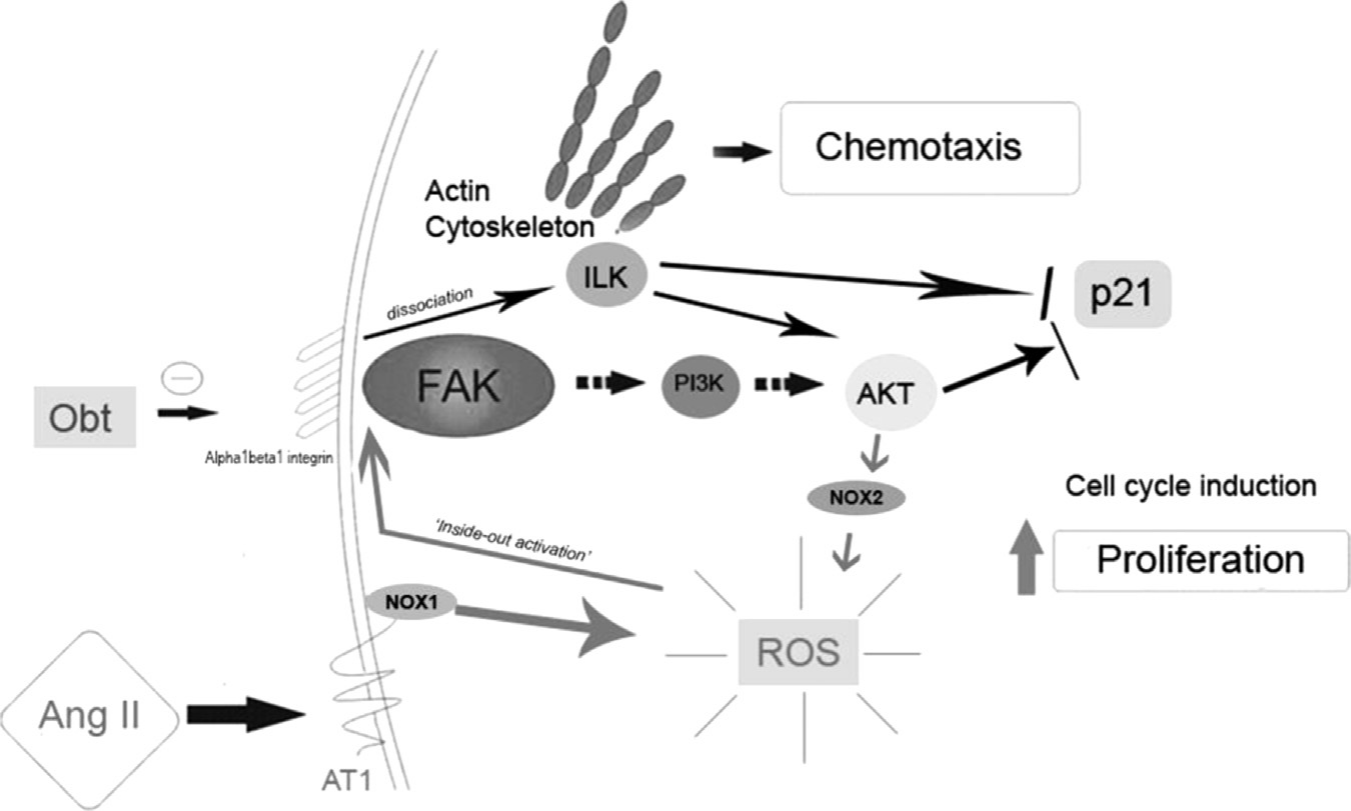
Proposed model. Ang II-induced ROS production is NOXs dependent and the initial ROS production induced by NOX1 leads to NOX2 activation, which sustains the effect. Alpha1beta1 integrin/ILK crosstalk is a link between NOX1 and NOX2 activation, which in turn is responsible for Ang II-induced VSMC migration and proliferation.

Taking together this data and Ref. [1], Ang II modulates signaling pathways involved in chemotaxis and proliferation in vascular smooth muscle cells. Ang II induces a rapid ROS production *via* NOX1. This initial and rapid ROS production activates alpha1beta1 integrin, which in turn activates FAK. Once activated, FAK dissociates to ILK, which activates AKT and induces p21 degradation. AKT promotes p47^*phox*^ engagement do NOX2 membrane subunit, allowing NOX2 complex activation, leading to ROS production that is responsible for cell migration and partially to cell proliferation (Fig. 10).

## 2. Experimental design, materials and methods

### 2.1 Cell culture

Primary VSMC were isolated from the aortic arch from naïve Wistar male rats using an explant method. In brief, rats were anesthetized and the thoracic aorta was harvested, opened longitudinally, scraped free of endothelium, cut into small pieces and treated for 30 min with collagenase. The endothelium-denuded tissue was placed with the luminal surface facing down on a 0.1% gelatin-coated flask with DMEM medium containing 10% FBS, 50 U/mL penicillin and 100 µg/mL streptomycin, and cultured at 37 °C/ 5% CO_2_ air atmosphere. After 7–14 days, the VSMC that outgrowth from the explants were trypsinized and used in different assays until passage three. These cells were positive stained for VSMC α-actin. This study was carried out in strict accordance with the recommendations in the Guide for the Care and Use of Laboratory Animals of the National Institutes of Health. The protocol was approved by the Committee on the Ethics of Animal Experimental of Universidade do Estado do Rio de Janeiro. (Permit number: CEUA/003/2013).

A7r5 VSMC, obtained from rat thoracic aorta (ATCC, Rockville, MD) were cultured in DMEM medium containing 10% FBS, 50 U/mL penicillin and 100 µg/mL streptomycin, and incubated at 37 °C in a 5% CO2 air atmosphere. The cells were dissociated with 0.1%/0.01% trypsin/EDTA and then seeded onto new culture flasks for a maximum of twelve passages.

### 2.2 mRNA silencing

A7r5 VSMC were transfected with either a non-targeting control siRNA (siRNA negative control # 1; Ambion, TX, USA) as a control for non-sequence-specific effects, or with an ILK (Silencer Pre designed siRNA ILK ID # 16708; Ambion), a NOX1-specific siRNA sequence (Silencer Predesigned siRNA NOX1 ID # 55585; Ambion) or a NOX2-specific siRNA sequence (Silencer Predesigned siRNA NOX2 ID # 647047; Ambion). Twenty µM of either the non-targeting or ILK, NOX1 or NOX2-specific duplex were diluted in 1 mL Opti-MEM I (Gibco,Grand Island, NY, USA) reduced serum medium and 1 mL of Lipofectamine 2000 (Ambion) diluted in Opti-MEM I was added. The mixture was gently mixed and allowed to incubate for 5 min at room temperature, being the final concentration of siRNA 10 µM. VSMC were grown in 6-well plate culture and when at 70% of confluence, RNAi duplex-Lipofectamine 2000 complexes in antibiotics and serum-free media was added. Twenty-four hours after transfection, attenuation of ILK, NOX1 or NOX2 expression was verified by Western blotting of cell lysates probed with antibody against ILK, NOX1 and NOX2.

### 2.3 Reactive oxygen species production

VSMC (5×10³ cells/well) were seeded in 96 wells plate overnight in DMEM medium containing 10% FBS. The cells were washed three times with PBS and the medium was replaced by DMEM containing 1% FCS for 1 h. VSMC were loaded with 10 µM CM-H2DCFDA (intracellular ROS) or with 10 µM MitoSox Red (mitochondrial-derived ROS) for 1 h and then washed to remove free probe. Cells were pretreated with 100 nM obtustatin or 10 µM apocynin for 15 min, and then incubated with Ang II 100 nM for 3 h at 37 °C. CM-H2DCFDA fluorescence was monitored at excitation and emission wavelengths of 495 and 530 nm, respectively and MitoSox Red fluorescence was monitored at excitation and emission wavelengths of 510 and 580 nm, respectively. Fluorescence was quantified using an EnVision® multilabel plate reader (Perkin-Elmer, Waltham, MA, USA). Slope intervals showed in tables were calculated by Δ*y*/Δ*x* of ROS production graph. Δ*y* is the amount of ROS production in determined interval time. Δ*x* is a time interval of 30 min.

### 2.4 Cell migration assay

For cell migration (chemotaxis) analysis *in vitro*, VSMC were seeded at a density of 2×105 cells/well in 48-well modified Boyden chambers containing a polycarbonate membrane (pore size, 10 µm; Neuroprobe, Inc., Gaithersburg, MD, USA), and incubated at 37 °C in a 5% CO_2_ atmosphere for 4 h. Some cell samples were treated with 10 µM Apocynin for 15 min before addition to the upper chamber (50 µL), and VSMC migrated toward 100 nM Ang II (in lower chamber). The cells that migrated to the lower membrane surface were fixed and stained using Wright-Giemsa stain and counted by light microscopy (400×), in an Olympus BX41 light microscope (Tokyo, Japan).

### 2.5 Cellular Extract

For whole cell extracts, VSMC were lysed in 50 mM HEPES, pH 6.4, 1 mM MgCl2, 10 mM EDTA, 1% Triton X-100, 1 µg/mL DNase, 0.5 µg/mL Rnase, 1 mM PMSF, 1 mM benzamidine, 1 µg/mL leupeptin and 1 µg/mL soybean trypsin inhibitor. For cell membrane isolation, VSMC were lysed in HES buffer (25 mM Hepes, pH 7.3, 1 mM EDTA, 0.25 M sucrose, aprotinin (10 µg/mL), leupeptin (10 µg/mL), pepstatin (2 µg/mL), and 1 mM PMSF). Lysates were ultracentrifuged at 40.000*g* for 1 h at 4 °C. Then, the supernatant was collected (cytosol fraction) and the pellet (membrane fraction) was ressuspended in HES buffer.

### 2.6 SDS-PAGE and immunoblotting

Total protein content in the cell extracts was determined by BCA method. Cell lysates were denatured in sample buffer (50 mM Tris·HCl, pH 6.8, 1% SDS, 5% 2-ME, 10% glycerol, and 0.001% bromophenol blue) and heated in boiling water for 3 min. Samples (30 µg total protein) were resolved by 12% SDS-PAGE and proteins transferred to polyvinylidinedifluoride membranes. Molecular weight standards were run in parallel. Membranes were blocked with Tween-TBS (TBS, 0.01% Tween 20; T-TBS) containing 1% BSA and probed with primary antibody (1:1000) overnight at 4 °C. Primary antibodies used in western analysis were anti-NOX1 cat: 131088 (Abcam, Cambridge MA, USA), anti-actin cat: 1501, anti-p47^*phox*^ cat: 07–497 (Millipore,Billerica, MA, USA), anti-GP91^*phox*^ (NOX2) cat: 20782, anti-NOX4 cat: 21860 (Santa Cruz Biotechnology, Santa Cruz, CA, USA). The membranes were rinsed with T-TBS and incubated for 1 h at room temperature with biotin-conjugated secondary antibody (1:10,000), followed by incubation with HRP-conjugated streptavidin (1:10,000). Immunoreactive proteins were visualized by the ECL detection. Western blotting signals were quantified by densitometry, using the ImageJ Software (NIH, Bethesda, MD, USA).

## Conflict of interest

The authors report no conflict of interest.

## Figures and Tables

**Table 1:** Alpha1beta1 modulates the second interval of Ang II-induced ROS production in VSMC. Slope interval was calculated by Δ*y*/Δ*x* of ROS production graph (see Fig. 1B in Ref. [1]). Δ*y* is the amount of ROS production in determined interval time. Δ*x* is a time interval of 30 min.

	**Control**		**Ang II**	
**Time interval (min)**	Untreated	+ Obtustatin	–	+ Obtustatin
0–30	0±0.07	0.06±0.1	0.73±0.01⁎	0.63±0.07⁎
30–60	0±0.05	0.06±0.1	0.5±0.14⁎	0.03±0.06#

Results are representative of three independent experiments. Data are expressed as means ± SD.

⁎ p<0.05 vs. untreated.

# p <0.05 vs the treated with Ang II.

**Table 2:** ILK modulates the second interval of Ang II-induced ROS production in VSMC. Slope interval was calculated by Δ*y*/Δ*x* of ROS production graph (see Figure 3B in Ref. [1]). Δ*y* is the amount of ROS production in determined interval time. Δ*x* is a time interval of 30 min.

	**Control**			**Ang II**		
**Time interval (min)**	siRNA scramble	siRNA NOX1	siRNA ILK	siRNA scramble	siRNA NOX1	siRNA ILK
0–30	0±0.11	0.03±0.13	0±0.08	0.9±0.11⁎	0.1±0.12#	0.83±0.12⁎
30–60	0±0.2	0±0.11	0.06±0.15	0.43±0.17⁎	0.1±0.17#	0.06±0.1#

Results are representative of three independent experiments. Data are expressed as means ± SD

⁎ p<0.05 vs. untreated.

# p <0.05 vs the treated with Ang II.
